# Fatal acute-on-chronic liver failure in amiodarone-related steatohepatitis: a case report

**DOI:** 10.1186/s12876-021-01632-9

**Published:** 2021-02-02

**Authors:** I.-J.u Wu, Jia-Huei Tsai, Cheng-Maw Ho

**Affiliations:** 1 Department of Emergency Medicine, National Taiwan University Hospital, Taipei, Taiwan; 2 Department of Pathology, National Taiwan University Hospital, Taipei, Taiwan; 3 Department of Surgery, National Taiwan University Hospital, 100 Taipei, Taiwan

**Keywords:** Amiodarone, Acute-on-chronic liver failure, Microabscess, Steatohepatitis, Case report

## Abstract

**Background:**

Amiodarone is an antiarrhythmic drug that has been recognized to induce hepatotoxicity. We report a case of acute-on-chronic liver failure (ACLF) in a patient who was receiving amiodarone for more than 2 years. The patient developed cirrhosis and suppurative microabscesses of the liver and died of progressive liver failure.

**Case presentation:**

A 69-year-old woman with risk factors for nonalcoholic fatty liver disease (NAFLD) was treated with oral amiodarone at a daily dose of 400 mg for more than 2 years, until she developed epigastralgia and vomiting. Initial laboratory findings included leukocytosis and elevated liver enzymes. Images of abdominal computed tomography scan revealed diffusely increased hepatic attenuation density (in contrast to decreased density in NAFLD), hepatomegaly, periportal edema, and ascites. Liver biopsy targeting the hotspot identified through positron emission tomography confirmed the diagnosis of amiodarone-associated chronic steatohepatitis and superimposed microabscesses. The patient died of progressive ACLF despite intensive supportive care.

**Conclusion:**

Accumulation of amiodarone can result in chronic liver disease and pose an additional risk of ACLF following infection.

## Background

Acute-on-chronic liver failure (ACLF) is a process of rapid deterioration in liver function occurring against a background of chronic liver disease characterized by multisystem organ failure, systemic inflammation, and high short-term mortality. Chronic liver disease is infrequently caused by potential hepatotoxic agents, such as amiodarone [1]. We report a rare case of ACLF caused by acute suppurative microabscesses superimposed on chronic drug-induced liver injury (DILI) associated with amiodarone.

## Case presentation

**Fig. 1:**
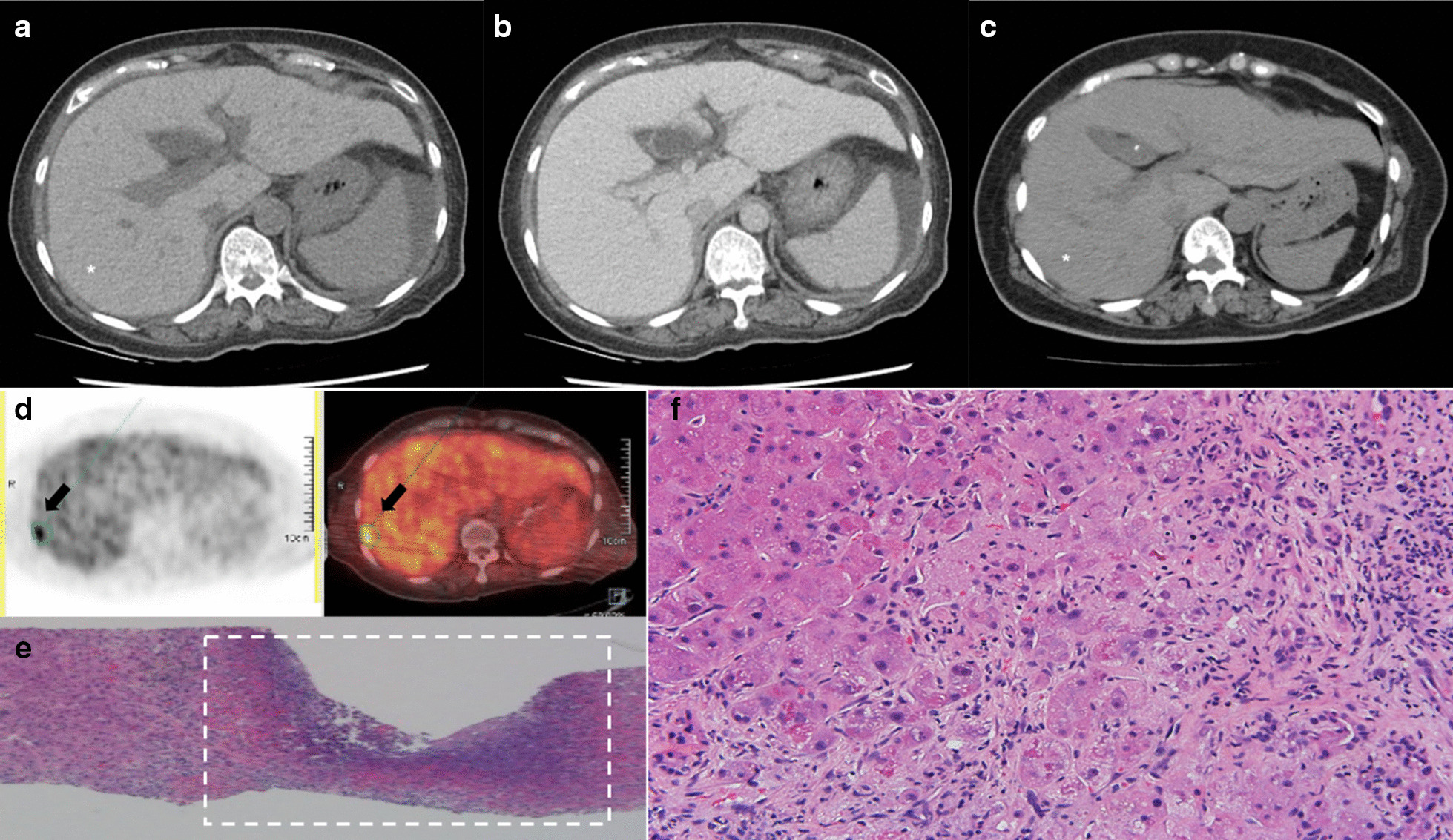
Development and diagnosis of ACLF. **a**–**c** Serial changes in hepatic signal intensity on CT imaging. High attenuation (90–100 HU) following (**a**) the use of amiodarone and (**b**) periportal edema in ACLF. **c** Low attenuation (50 HU) of liver parenchyma 2 years prior to exposure to amiodarone. **d** FDG-PET scanning demonstrated focal hypermetabolic hepatic lesions in both lobes, with a maximum standardized uptake value of 8.8 (arrow). **e**, **f** Histopathological examination showed (**e**) suppurative microabscesses (dotted frame) and (**f**) numerous Mallory-Denk bodies in the periseptal hepatocytes

**Fig. 2:**
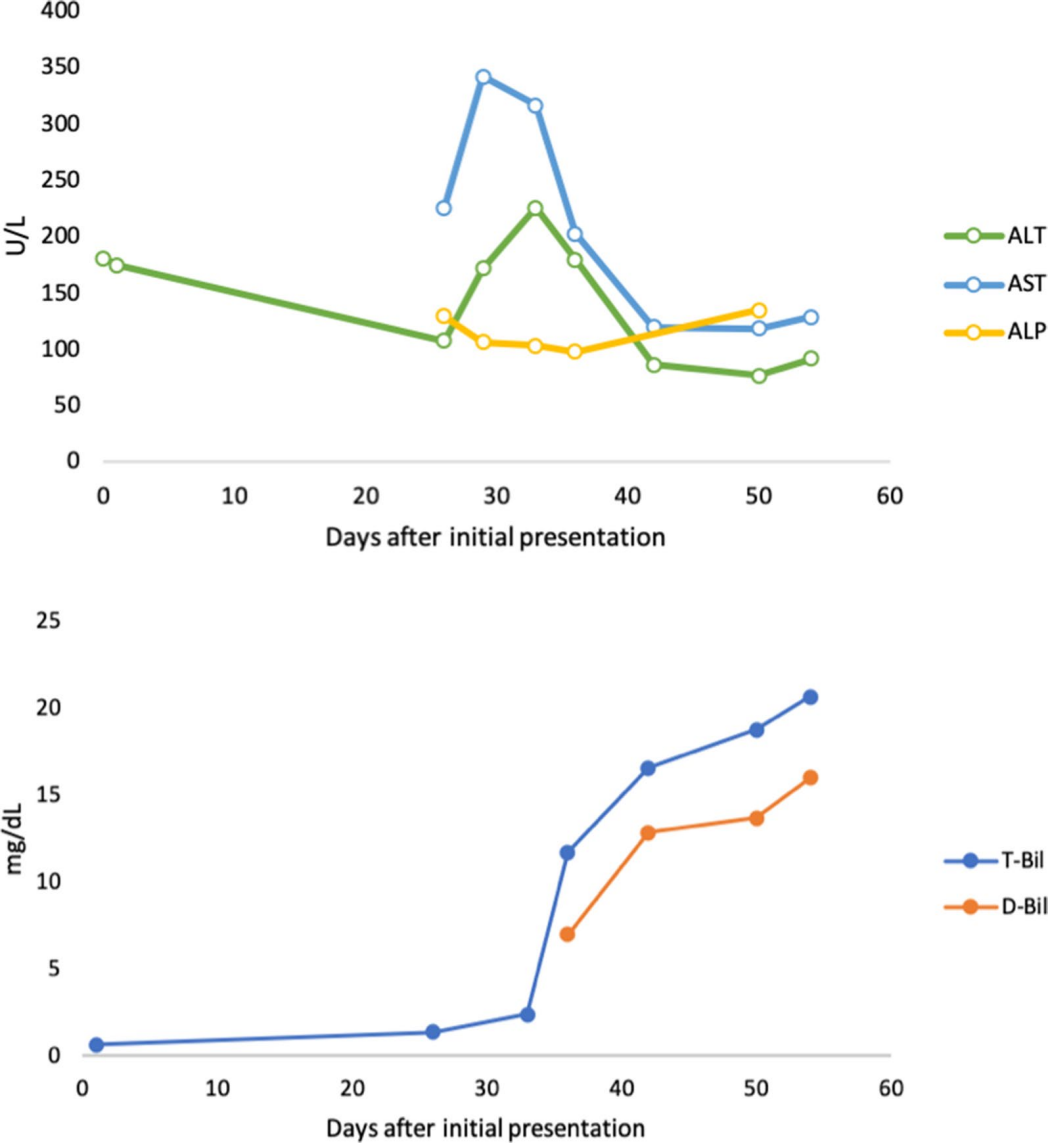
Trends of liver function tests since the initial presentation. AST, aspartate transaminase; ALT, alanine transaminase; ALP, alkaline phosphatase; T-Bil, total bilirubin; D-Bil, direct bilirubin

**Fig. 3:**
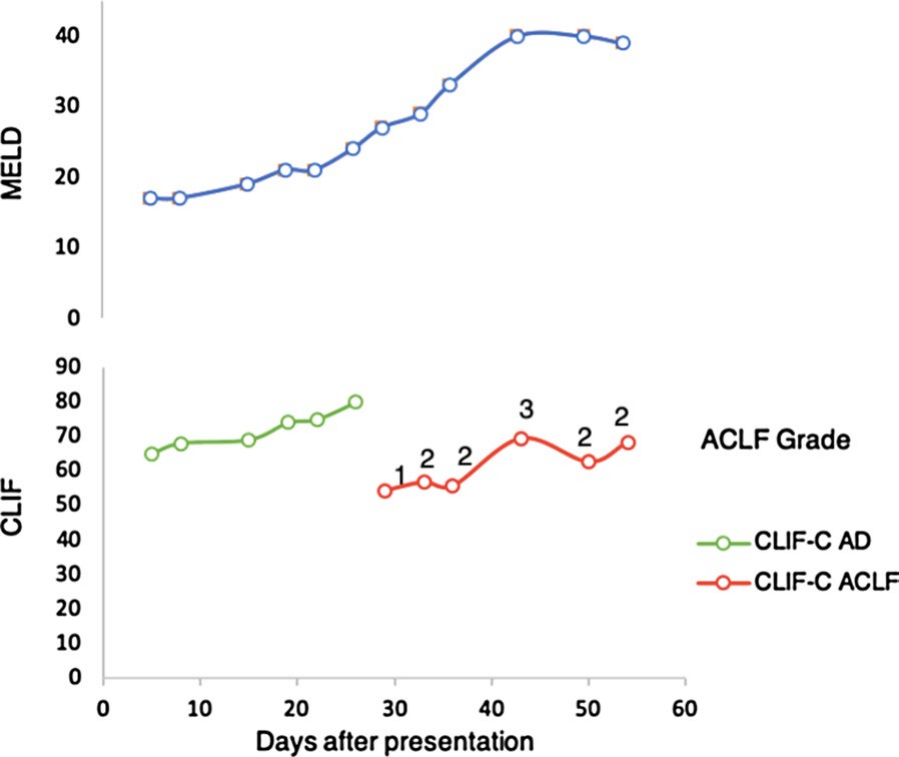
Trend of severity scores since the initial presentation. ACLF, acute-on-chronic liver failure; AD, acute decompensation; CLIF-C, Chronic Liver Failure Consortium; MELD, Model for End-stage Liver Disease

A 69-year-old woman was admitted to our hospital for postprandial epigastralgia and vomiting for 2 weeks. She had a history of diabetes mellitus and hyperlipidemia, and she had undergone bypass surgery for coronary arterial disease 2.5 years previously. She had no alcohol consumption. Initial evaluation revealed distended abdomen, epigastric tenderness, neutrophilic leukocytosis (17.3 k/μL), elevated C-reactive protein, pyuria, liver panel abnormalities (i.e., hepatitis and conjugated hyperbilirubinemia), and coagulopathy. At presentation, the serum levels of aspartate transaminase (AST), alanine transaminase (ALT), total bilirubin, direct bilirubin, albumin, and international normalized ratio of prothrombin time were 226 U/L, 108 U/L, 11.72 mg/dL, 6.97 mg/dL, 2.1 g/dL, and 1.44, respectively. Two months before this admission, the serum levels of AST, ALT, total bilirubin, and direct bilirubin were 144 U/L, 131U/L, 0.64 mg/dL, and 0.21 mg/dL, respectively. The AST to platelet ratio index (APRI) score was 2.04, an increase from 1.23 in 2 years. A hepatopathy screening including viral hepatitis (hepatitis B, C, D, and E), auto-immune and hereditary liver disease was negative, except a document of remote hepatitis A infection. Computed tomography (CT) scanning disclosed hepatomegaly, periportal edema, and ascites (Fig. 1a, b). Notably, a diffuse increase in hepatic attenuation density, a hallmark of iron or amiodarone deposition, was observed. Amiodarone had been prescribed at a daily dose of 400 mg (estimated total cumulative dose exposure of 360 g) since the bypass surgery for tachyarrhythmia. Liver attenuation was approximately 50 Hounsfield units (HU) before exposure to amiodarone (Fig. 1c) and had increased one-fold by the following year. Amiodarone was discontinued upon admission. Broad-spectrum antibiotics were used to treat a documented vancomycin-resistant enterococcal urinary tract infection and other potential occult infections without significant clinical improvement. Leukocytosis persisted and a fluorodeoxyglucose (FDG)-positron emission tomography (PET) scan 3 weeks after admission revealed multiple areas of active inflammation within the liver (Fig. 1d). The pathological findings of a targeted biopsy of the largest hot spot (Fig. 1d, arrow) in the FDG-PET scan revealed multifocal necrotizing suppurative microabscesses accompanied by aggregates of granulation tissue and histiocytes (Fig. 1e). No pathogen was observed on acid-fast, periodic-acid-Schiff, and Grocott's methenamine silver staining. Moreover, the liver parenchyma demonstrated cirrhosis and regenerative nodules associated with prominent periseptal ballooned hepatocytes containing numerous Mallory-Denk bodies (Fig. 1f). The changes of selective liver panels since the initial presentation were illustrated in Fig. 2. A rare presentation of ACLF was confirmed based on the findings of amiodarone-associated chronic steatohepatitis and superimposed microabscesses. The patient’s Chronic Liver Failure Consortium (CLIF-C) ACLF score was calculated to be 69.4, and the model for the end-stage liver disease (MELD) score was high (> 30), indicating an unfavorable outcome [2] (Fig. 3). The patient’s clinical condition deteriorated, and she died of progressive liver and renal failure.

## Discussion and conclusions

Amiodarone accounts for 1–3% of all DILIs, most commonly exhibiting a hepatocellular injury pattern, particularly in patients with older age, lower body surface area, and dyslipidemia [3]. Elevation of liver enzymes following high-dose intravenous infusion can occur and is mediated through direct hepatotoxicity, which is usually transient and self-reversible; by contrast, liver cirrhosis due to chronic use of low-dose amiodarone is caused by nonallergic idiosyncratic reactions of accumulation-related injury [4]. Amiodarone could induce lysosomal phospholipidosis, resulting in formation of Mallory-Denk bodies in the liver and inhibiting phagocytosis, the latter of which might impair macrophage function and contribute to ACLF progression as observed in the present case [5]. Steatohepatitis associated with amiodarone, through inhibition of the β-oxidation of free fatty acid in the mitochondria [4], exhibits high HU values, in contrast to hypointensity (low HU values) of the liver in nonalcoholic steatohepatitis associated with metabolic syndrome. However, additional hepatotoxicity by amiodarone on top of steatohepatitis related to her metabolic syndrome/insulin resistance is the most likely scenario of her chronic liver disease based on the above negative hepatopathy panel [6].

With the development of toxicity, no definite treatment exists for amiodarone-induced liver injury apart from drug discontinuation, and the mortality rate is as high as 60% at 5 months. The cumulative dose in our patient was 360 g over 2.5 years, close to the reported dose deemed sufficient to induce liver cirrhosis [7]. Together with acute inflammation and possibly macrophage dysfunction, we propose that amiodarone serves as a crucial contributor to the development of ACLF.

In conclusion, accumulation of amiodarone can result in chronic liver disease and pose an additional risk of developing ACLF following infections.

## Data Availability

Not applicable.

## Abbreviations

ACLF: acute-on-chronic liver failure
ALT: alanine transaminase;
AST: aspartate transaminase
CLIF-C: chronic liver failure consortium
CT: computed tomography
DILI: drug-induced liver injury
FDG: fluorodeoxyglucose
HU: hounsfield unit
MELD: model for end-stage liver disease
PET: position emission tomography
